# On the Diagnosis of Chronic Ovarian Tumors

**Published:** 1852-09

**Authors:** E. J. Tilt

**Affiliations:** Senior Physician to the Farringdon General Dispensary and Lying-in Charity, and to the Paddington Free Dispensary for Diseases of Women and Children


### SURGERY.

*On the Diagnosis of Chronic Ovarian Tumors.* By E. J. TILT, M. D., Senior Physician to the Farringdon General Dispensary and Lying-in Charity, and to the Paddington Free Dispensary for Diseases of Women and Children.—At different times we have published, in the *Lancet* and in the *London Medical Gazette,* a series of papers on the "Pathological Anatomy of Chronic Ovarian Growths, and on their

Cause, Progress, and Termination." We now propose treating of the diagnosis of these tumors, and feel no little hesitation in beginning a chapter which must be the history of the mistakes daily committed, even by the most enlightened.

The difficulties of the diagnosis of chronic ovarian tumors arise from the form and nature of the cavity in which the abdominal viscera are contained. The more easily defined limits of other organs render the diagnosis of these diseases comparatively easy; but it was necessary, for the health of the body, and to economise the space allotted to the abdominal viscera, that they should have no fixed limits, in order that they might, in their periodical ebb and flow, appropriate each according to its peculiar wants more or less of the same cavity. This community of habitation entails a certain obscurity of position, and gives rise to a corresponding obscurity of diagnosis of the diseases of the abdominal organs. With other organs we find that any obscurity of diagnosis which might have existed in the origin, diminishes as the disease progresses, but the certainty of the diagnosis of ovarian tumors does not increase in proportion to their dimensions, because each of the neighboring viscera—womb, liver, spleen, kidney, and bladder, are susceptible of attaining to a similar bulk, and these organs must, therefore, be believed to be in a healthy state by the medical attendant, before he can diagnose an ovarian tumor.

Another difficulty of abdominal diagnosis arises from the fact, that the abdominal cavity, more than any other, harbors not unfrequently tumors of stupendous size, which have no other origin than some minute sub-peritoneal cell. In such cases we need not be surprised that a tumor, which belongs to no organ, should be, by different medical men, attributed in turn to a swelling of each of the abdominal organs in the vicinity of which it is developed.

It may be thought that we exaggerate the uncertainty of abdominal diagnosis, but we do not hesitate to say that it is more difficult to diagnose many complaints of the organs contained in this cavity than those of the chest or the head. Medical men have sometimes brought us patients said to be affected with ovarian dropsy, but who were in reality suffering from fibrous tumors of the womb, chronic peritonitis, or even *embonpoint*, particularly when the patients have arrived at the age of cessation of menstruation. All those who are engaged in active practice must be able to recall similar proofs of the great imperfection of abdominal diagnosis, and the operations of ovariotomy furnish us with wholesale examples of the errors of diagnosis made by the most eminent in our profession; errors made by men who have specially studied ovarian disease, and the individual cases of that disease which they attempted to cure radically by an operation, on the value of which they staked their medical reputation. Dr. Dolhoff, at Magdebourg, had studied his patient's case for a year; many eminent men who had seen the patient with him were convinced of the existence of a tumor, and still none was found when the abdomen was opened. The best opinions of Edinburgh confirmed Lizars in his opinion, that a patient of his was affected with an ovarian tumor. An operation was performed, but no tumor was found. We learn from Dr. Atlee's valuable table, that out of 179 cases of attempted ovariotomy, the operation could not be completed in 34. This means in plain English, that in 34 out of the 179 the surgeons made an unfortunate error of diagnosis. In 17 of these cases other important diseases existed, which would have contraindicated ovariotomy, but as these were not detected by the surgeon, the 17 operations were performed, and occasioned the death of 14 of the patients. Adhesions of the tumors to neighboring organs are also considerable complications when they are extensive, as was the case in 62 operations out of the above mentioned 179. In other words, these 62 cases were also errors in diagnosis; for if the surgeon had suspected the existence of these adhesions, he would, in most of the cases, have declined operating, well knowing that this complication increased the difficulties of the operation, increased the ratio of its mortality, and sometimes rendered it impossible. In six cases, says Dr. Atlee, (but two others are recorded,) no tumor was found, so that in these patients the abdomen was opened, it was examined for a tumor, and none was found, and then the abdomen was sewn up. The 179 operations of ovariotomy thus afford us proof of 121 errors of diagnosis.

While, however, admitting the imperfection of our diagnosis of abdominal diseases, we must not omit mentioning that some of our means of diagnosis have lately been improved, and that others have recently been added, which, if judiciously used, permit us to anticipate a diminution of what has been well termed "ovarian perplexities."

In Professor Piorry's hands Avenbrugger's discovery has been proved to be equally serviceable in detecting diseases of the abdomen as those of the thorax. Kergaredec applied his uncle's stethoscope to the abdomen, and taught us to distinguish a sound, by which, if distinctly heard, we can be confident of pregnancy. And later still, the head of the obstetric department of our profession in Scotland, Dr. Simpson, has given us a very important instrument of diagnosis—the uterine sound, by means of which we are generally able to eliminate the uterine element from very complicated problems of abdominal morbid structure. This instrument has indeed been inveighed against, with considerable force, by some who have been peculiarly struck by cases wherein the instrument was injudiciously employed, but as a help to diagnose abdominal disease, it is undoubtedly of great utility, a conviction expressed with the greater pleasure, because we differ in some other points of practice with its illustrious inventor.

Having shown the difficulties of abdominal diagnosis, having pointed out the reason of these difficulties, and glanced at some of the means by which they may be diminished, we shall now proceed systematically in our attempt to unravel the obscurity which we deplore. Our idea of diagnosis is to distinguish some peculiar disease from all those with which it may be confounded, we shall then proceed to pass in review, one after the other, all the diseased organs with which ovarian tumors have been confounded, or, in other words, all the abdominal viscera.

Should the reader find that we are tedious in the inquiry, let him remember that in learning to detect ovarian tumors he unavoidably learns to discover the other tumors or diseases of the structure which so frequently occur in the abdomen, and that he will thus acquire a knowledge of abdominal disease, the obscurest and the most important part of medical diagnosis, for the viscera of nutrition are those principally affected in all diseases. Let him remember that the rarity of any peculiar disease should not be an unconquerable obstacle to the sagacity of the practitioner, who knows how to *seek* for the signs of disease and how to interpret them. Acquainted with the anatomy of the visceral organs, and conversant with their functions, the medical attendant should be ready to interpret those rare cases which, in the course of practice, may perhaps "turn up" to-morrow.

Chronic ovarian tumors have been confounded with ovaritis, on account of the extraordinary size of the acutely-inflamed ovary, but the rapidity of the development of the tumor, and the fever by which it is attended, will not permit the practitioner to be easily deceived; and the rapidity of the march of the disease will soon show the disease to be acute. On account of the acute march of an ovarian cyst, it may, however, be confounded with ovaritis. In the *London Medical Gazette* we have related an interesting example of this; the fever was such as attends acute disease, the iliac pain was intense, the swelling rapid, and we should have remained convinced that the tumor was of a purulent nature, if an exploratory puncture had not been made, and a transparent albuminous fluid withdrawn. A careful examination of the patient could alone prove that, in such cases, the ovary is affected, the knowledge of the nature of the tumor being left to be determined by the subsequent history of the case, a delay little to be regretted, as in both cases the indications would be the same, that is to say, to arrest the progress of inflammation by bloodletting and mercury. Ovarian tumors have been confounded with pelvic tumors, sanguineous or purulent, developed in the recto-vaginal pouch. By means of the uterine sound it is easy to eliminate the uterus from the morbid problem, but it is sometimes difficult to distinguish an incipient ovarian from a pelvic tumor, in cases similar to those described by Dr. Seymour, where the falling of the ovarian tumor into the recto-vaginal pouch gives rise to excessive pain. A few days will generally suffice to clear up the difficulties of diagnosis, for while the distressing symptoms attendant on the position of the tumor diminish, or at least do not increase, the constitutional symptoms will increase if the tumor be purulent, and it will most likely point towards the vagina, the rectum, or the skin, in the inguinal region. In very rare cases, however, it may happen that a purulent tumor attains a large size in the recto-vaginal pouch, without the difficulty of diagnosis being diminished, as when they imitate those ovarian tumors which are quite round, central, painful, and bulge into the vagina. In a very interesting case, recorded at great length, by Dr. Camus, (*Revue Medicale,* 1838,) the patient would certainly have been considered to be suffering from ovarian dropsy if it had not been known that the disease originated in the culpable introduction of an instrument into the womb to procure abortion. The tumor took several months to attain the umbilicus. In this case an obscurity in the diagnosis was not much to be regretted as it did not increase the difficulty of the treatment. The indications were similar in the event of either disease, and a vaginal puncture of the bulging portion which did cure the abscess, might also have radically cured the ovarian cyst, as has happened in many an instance.

*Sanguineous pelvic tumors* are more likely to be confounded with incipient ovarian tumors, and as they have not been described by classic authors and have been but lately investigated by French pathologists, it may be well to glance at their history. In the midst of menstrual disorders, consisting in the flow being more or less abundant and painful than usual, intense hypogastric pain arises with fever, which lasts a few days, then ensues a dull heavy bearing down pain in the hypogastric region, and a large collection of blood forms in the recto-vaginal pouch, which, by pressing the rectum in and against the concavity of the sacrum, gives rise to obstinate constipation, whilst by pressing the urethra against the pubes, the same tumor causes a difficulty or impossibility of passing water. At first fluctuation may be easily detected in this tumor, but when the blood coagulates it becomes hard, and in one instance was mistaken by Malgaigne for a fibrous tumor of the womb. The tumor often disappears by resolution, or else it may open and discharge its contents in the vagina or the rectum.

These details are sufficient to show that when ovarian cysts are so confined by false membranes, as to be developed in the recto-vaginal pouch, it may be difficult to distinguish them from sanguineous pelvic tumors, but the more rapid growth of the latter and their early fluctuation, enable the practitioner to form a correct opinion, and time will give certainty to the diagnosis.—*Prov. Med. and Surg. Journ.*

